# Nutrient Dynamics of Estuarine Invertebrates Are Shaped by Feeding Guild Rather than Seasonal River Flow

**DOI:** 10.1371/journal.pone.0137417

**Published:** 2015-09-09

**Authors:** Kelly Ortega-Cisneros, Ursula M. Scharler

**Affiliations:** School of Life Sciences, Westville Campus, University of KwaZulu-Natal, Durban, South Africa; Dauphin Island Sea Lab, UNITED STATES

## Abstract

This study aimed to determine the variability of carbon and nitrogen elemental content, stoichiometry and diet proportions of invertebrates in two sub-tropical estuaries in South Africa experiencing seasonal changes in rainfall and river inflow. The elemental ratios and stable isotopes of abiotic sources, zooplankton and macrozoobenthos taxa were analyzed over a dry/wet seasonal cycle. Nutrient content (C, N) and stoichiometry of suspended particulate matter exhibited significant spatio-temporal variations in both estuaries, which were explained by the variability in river inflow. Sediment particulate matter (%C, %N and C:N) was also influenced by the variability in river flow but to a lesser extent. The nutrient content and ratios of the analyzed invertebrates did not significantly vary among seasons with the exception of the copepod *Pseudodiaptomus* spp. (C:N) and the tanaid *Apseudes digitalis* (%N, C:N). These changes did not track the seasonal variations of the suspended or sediment particulate matter. Our results suggest that invertebrates managed to maintain their stoichiometry independent of the seasonality in river flow. A significant variability in nitrogen content among estuarine invertebrates was recorded, with highest % N recorded from predators and lowest %N from detritivores. Due to the otherwise general lack of seasonal differences in elemental content and stoichiometry, feeding guild was a major factor shaping the nutrient dynamics of the estuarine invertebrates. The nutrient richer suspended particulate matter was the preferred food source over sediment particulate matter for most invertebrate consumers in many, but not all seasons. The most distinct preference for suspended POM as a food source was apparent from the temporarily open/closed system after the estuary had breached, highlighting the importance of river flow as a driver of invertebrate nutrient dynamics under extreme events conditions. Moreover, our data showed that estuarine invertebrates concentrated C and N between 10–100 fold from trophic level I (POM) to trophic level II (detritivores/deposit feeders) and thus highlighted their importance not only as links to higher trophic level organisms in the food web, but also as providers of a stoichiometrically homeostatic food source for such consumers. As climate change scenarios for the east coast of South Africa predict increased rainfall as a higher number of rainy days and days with higher rainfall, our results suggest that future changes in rainfall and river inflow will have measurable effects on the nutrient content and stoichiometry of food sources and possibly also in estuarine consumers.

## Introduction

Ecological stoichiometry focuses on studying the balance of elements in ecological interactions and processes [1]. As such it has helped to provide a better understanding of ecosystem functioning and dynamics, because it allows comparisons among organisms and ecosystems by expressing their composition in biogeochemical terms [1]. Several principles have been developed in ecological stoichiometry theory, which aim to explain the characteristics of elemental composition and ratios in organisms. Stoichiometric homeostasis is one of these principles and refers to the capacity of an organism to maintain a constant chemical composition regardless of variations in the composition of its resource nutrient content [1]. Homeostasis can be either weak, when an organism changes its elemental composition according to that of its resource (i.e. autotrophs are assumed to have a weak homeostasis), or strict, when the elemental composition is kept constant despite the variability in the elemental ratios of food sources (i.e. most heterotrophs) [2–4]. Later studies have however indicated that heterotrophs may not be as strictly homeostatic as assumed [5–7]. Persson et al. [8] analyzed 132 datasets from published studies to test the generality of the strict homeostasis assumption; they found that autotrophs were generally less homeostatic than heterotrophs, but that heterotrophic species differed in their degree of homeostatic regulation. Moreover, these authors suggest that the degree of homeostasis is strongly influenced by both environmental and physiological factors. Stoichiometry imbalances are caused by the differences in elemental composition between resources (i.e. nutrient poor, high C:nutrient ratio) and consumers (i.e. nutrient rich, low C:nutrient ratio), which use diverse physiological mechanisms to compensate for the elemental imbalance [9,10]. Large elemental imbalances between primary food sources and consumers can modify the growth, reproduction and nutrient release of an organism, with consequences at the community and ecosystem level [3,9,11].

Estuaries are highly productive ecosystems and provide important ecosystem services to society, i.e. they are nursery areas for many fish and invertebrate species, and it is therefore of importance to understand their functioning and nutrient dynamics [12]. Historically, the majority of stoichiometry studies in estuaries focused on dissolved inorganic nutrients and the nutrients contained in particulate organic matter, with estuarine taxa receiving less attention. The fitness of organisms however is determined by both the absolute amount of nutrients contained in a food source as well as their stoichiometry [1]. Freshwater inflow to estuaries is a crucial factor controlling nutrient concentrations, species composition, abundance and biomass of estuarine communities (e.g. [13,14]) and thus may also influence diet proportions of organisms due to variable abundance of diet items. Particulate organic matter (POM) is a basic, and highly abundant food source in estuaries, important for dietary requirements of deposit and detritus feeders, and supplementing omnivores diet. As such, detritivory on POM in general exceeds that of herbivory in South African estuaries (e.g. [15,16,17]). A number of authors have documented variations in the abiotic and biotic components of estuaries in South Africa as a result of changes in river flow, especially in terms of open/closed phases in temporarily open/closed systems (e.g. [18,19,20]). As freshwater is a scarce resource in many regions globally, an understanding of the response of nutrient dynamics in estuarine food webs to variable freshwater input is critical. In South Africa, rainfall patterns vary markedly across the country and follow a seasonal pattern [21]. This study was conducted in the subtropical region of South Africa, specifically the KwaZulu-Natal coast, characterized by a rainy season (October–April) with strong river inflow to estuaries and a dry season (May—September) with a comparatively lower average rainfall and often negligible river inflow to estuaries. The seasonal variability in rainfall and river inflow have been found to produce significant spatio-temporal changes in the estuarine communities of this region [19,20]. It was thus hypothesized that the seasonal variability in rainfall and river inflow may also produce significant spatio-temporal changes in the elemental content, stoichiometry and the utilization of food sources of estuarine planktonic and benthic communities.

We therefore aim to provide an insight into the variability of elemental content, stoichiometry and utilization of food sources in two sub-tropical estuaries by focusing on the influence of seasonal changes in rainfall and river inflow on abiotic sources, zooplankton and macrozoobenthos taxa. We tested the following hypothesis: 1) the % C, % N, and C:N ratio of suspended and sediment particulate organic matter vary among seasons and stations in two subtropical estuaries, 2) the C:N ratio of the zooplankton and macrozoobenthos is homeostatic (constant) across seasons, 3) the zooplankton and benthic invertebrates have different % C, % N, and C:N ratio in these two subtropical estuaries (among-taxa variability) and 4) the diet composition of estuarine invertebrates in these two systems vary through time following the variability of river inflow.

## Materials and Methods

### Study sites

**Fig 1:**
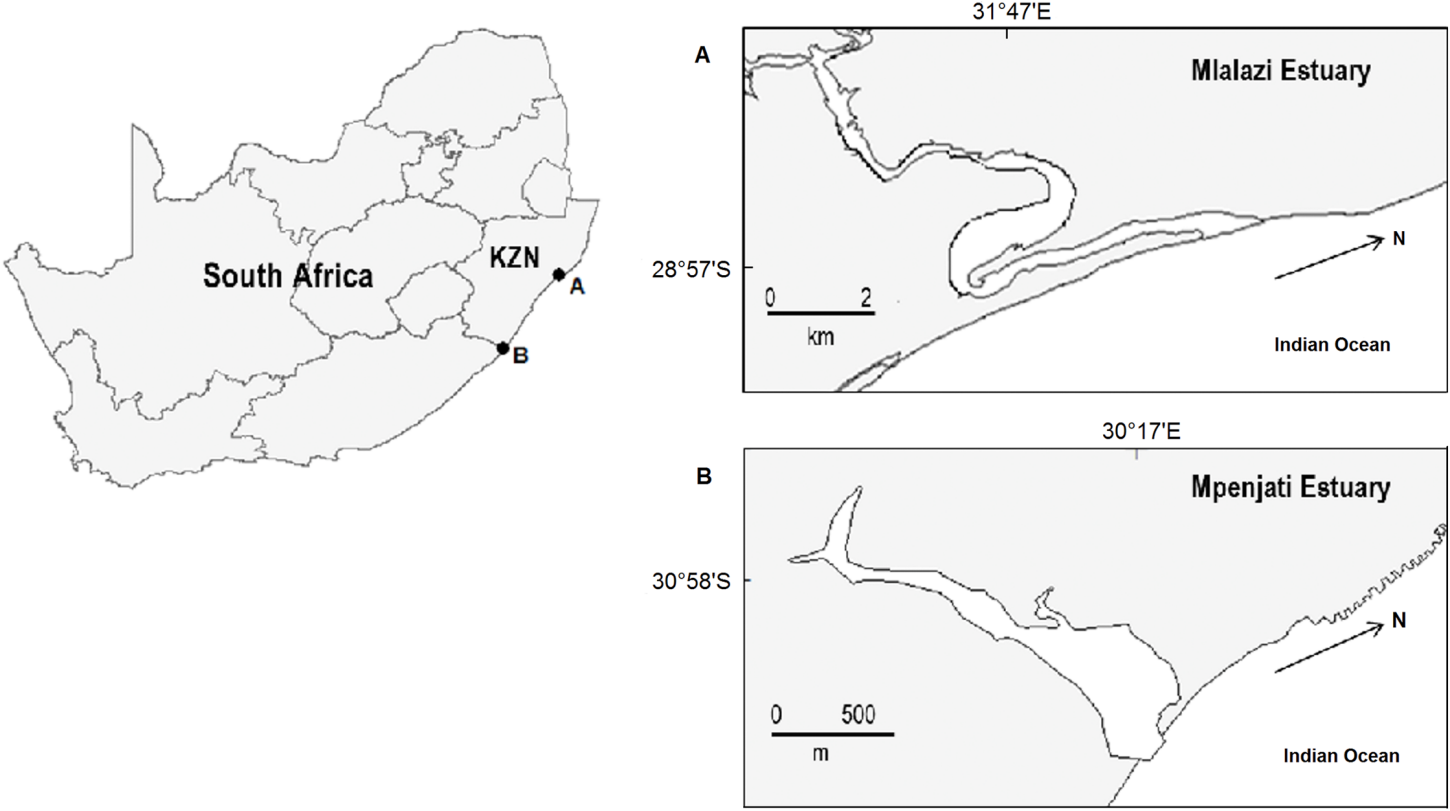
Map of the study areas (Mlalazi and Mpenjati estuaries) on the east coast of South Africa. Dots represent sampling stations.

The KwaZulu-Natal coast, the focus of this study, comprises the northeast coast of South Africa and the largest part of its subtropical biogeographical region (Fig 1). The two study estuaries, the Mlalazi and Mpenjati, are of the permanently open and temporarily open/closed type respectively. Temporarily open/closed estuaries predominate in South Africa (~ 65%), whereas only about 13% of South Africa’s estuaries have a permanent connection to the sea [22,23]. The Mlalazi Estuary (28° 56'42'' S; 31°48' 58'' E) has a catchment area of approximately 492 km^2^ and an estuarine area of 0.96 km^2^ [24]. Subsistence farming, sugar cane and commercial forestry accounts for approximately 46% of the catchment usage, with 53% of the catchment considered not degraded and 1% urban.

The temporarily open/closed Mpenjati Estuary (30°58′21''S, 30°17'02′'E) has a catchment area of approximately 101 km^2^ and an estuarine area of 0.12 km^2^ [24]. The Mpenjati Estuary experienced two mouth openings during the study period, it was connected to the sea from the end of October 2010 to mid-April 2011 and from mid-May to July 2011 during an abnormally wet winter. Most of the catchment is used for banana and sugar cane farming. A waste water treatment plant is located alongside the upper reaches of the estuary and discharges treated water into the system (~ 0.015 million m^3^ per month) [25], and the discharge is monitored to ensure water quality parameters are within acceptable limits for human contact. Both systems are part of nature reserves and considered to be in good condition [22].

### Sampling procedure

Biological and environmental samples were collected every three months from the Mlalazi and Mpenjati estuaries between September 2010 and May 2011. Three stations were sampled in the Mpenjati Estuary and four stations in the Mlalazi Estuary (Fig 1), with the difference in number of stations being based primarily on the differences in estuary length. The stations were located so that representative spatial samples were collected in the upper, middle and lower reaches of the respective estuaries. Sampling was conducted during the day, September 2010 was representative of the dry season, November 2010 and February 2011 of the wet season, and May 2011 again of the dry season. The latter turned out to be abnormally wet and caused breaching of the Mpenjati Estuary in a season when it was expected to be closed.

Physico-chemical measurements (salinity, temperature, pH, and dissolved oxygen) were taken at the sub-surface and bottom of the water column using a YSI 6920 water quality logger at each station. Rainfall data were obtained from the South African Sugarcane Research Institute (SASRI) website for the catchments of the Mlalazi (Station 478—Empangeni) and Mpenjati (Station 101 –Southbroom) estuaries. Triplicate water samples for the determination of suspended particulate matter (susPOM) were collected in acid washed plastic bottles from ca. 50 cm depth at each station and subsequently stored in the dark on ice. Sediment particulate matter (sedPOM) was determined by collecting three sediment samples at each station to a depth of 2.5 cm using a twin-corer (internal diameter 2 cm).

Zooplankton samples were collected using a hyperbenthic sled with a 200 μm mesh plankton net. Two replicates were collected at each station and then preserved in 5% formalin with Rose Bengal. Macrozoobenthos samples were collected using a Zabalocki-type Ekman grab (September 2010) and a van Veen grab (for the rest of the study) at each station. Three replicates were collected at each station, each consisting of three grabs. Macrozoobenthos samples were filtered using a 500 μm sieve and the contents preserved in 5% formalin using Rose Bengal. Organisms were preserved after collection for identification and enumeration, therefore gut evacuation was not conducted before analyses.

Zooplankton samples were suspended in 1 to 5 L solutions, depending on the concentration of organisms. From each solution, three 20 ml subsamples were withdrawn at mid-depth, while stirring continuously to ensure homogenous suspension. Zooplankton and macrozoobenthos organisms were identified and counted using a dissecting microscope. Zooplankton and macrozoobenthos samples were processed within 3 months of collection, and taxa identified to the lowest possible taxonomic level.

### Elemental analysis

Seston samples, for the analysis of suspended particulate organic carbon and nitrogen, were pre-filtered through a 200 μm mesh to avoid debris or larger organisms to be included on the samples. This filtrate was then filtered through a 0.72 μm pre-combusted and pre-weighted GF/F filter, and the filters containing seston were dried at 60°C for 24 h. Afterwards, the filters were stored in aluminium foil bags within a desiccator until further analysis. To determine sediment nutrient content, sediment samples (~ 8 g) were dried at 60°C for 48 h, ground to ensure homogenization and then acidified using 2% HCl solution to remove carbonates. Afterwards, sediment samples were rinsed with Milli-Q water and then dried again at 60°C for 48h.

After oven-drying at 60° for 48 h, zooplankton and macrozoobenthos samples were placed in 1.5 ml polypropylene microcentrifuge tubes. For the elemental composition analysis of zooplankton taxa, one replicate consisted of three up to several hundred individuals of each taxa depending on the size and weight of the organisms. For macrozoobenthos samples, three to 100 individuals comprised one replicate sample. All zooplankton and macrozoobenthos samples were crushed and homogenized with a glass rod within the centrifuge tubes to avoid loss of material during the homogenisation process. Macrozoobenthic organisms containing CaCO_3_ were acidified (i.e. crabs) with 2% HCl to remove carbonates. The drop-by-drop acidification technique was used, 2% HCl was added to the samples until all CaCO_3_ was removed and no more bubbles were evident [26]. Thereafter, the samples were washed in distilled water to remove acid and once again oven-dried at 60°C for 48h.

Between 5 and 150 mg was required for the elemental analysis of sediment samples, and from 0.5 to 1.2 mg for C and N analysis of zooplankton and macrozoobenthos taxa. Ideally, three replicates (each composed of several individuals) were analyzed for each taxon, sampling station and sampling event, except when a given taxon was not present on a particular sampling date or when the material available was insufficient for analysis. Since not all taxa occurred in all samples at all sampling events, the number and taxa used for nutrient analysis differed among sampling events. A total of 18 estuarine taxa were analyzed in this study.

Carbon and nitrogen content (% of dry weight), C:N ratios and isotope signatures were obtained for all samples using an ANCA-SL (Automated Nitrogen Carbon Analyzer—Solids and Liquids) elemental analyzer coupled to a Europa Scientific 20–20 isotope ratio mass spectrometer (IRMS) (Europa Scientific Limited, Crewe, England). Samples were analyzed at Isoenvironmental cc, Rhodes University, Grahamstown. Beet sugar, ammonium sulphate and five certified protein standard (casein) were used as international standards and calibrated against International Atomic Energy Agency [IAEA] standards IAEA-CH-6 and IAEA-N-1. The analytical precision of the IRMS was 0.21 ‰ for ^15^N/^14^N and 0.17 ‰ for ^13^C/^12^C. Results were calculated and expressed in the standard delta notation as:

δC13(‰)=([Rsample/Rstandard]−1)×1000

δN15(‰)=([Rsample/Rstandard]−1)×1000

where R is the ratio of ^15^N:^14^N or ^13^C:^12^C in the sample (R _sample_) and in the standard (R _standard_).

### Data analysis

In order to determine spatio-temporal variations in the %C, %N, C:N ratio and isotope signatures of suspended and sediment particulate matter in each estuary, two-way ANOVA were conducted using seasons and stations as factors. Several one-way ANOVAs were conducted to test for significant seasonal differences in the %C, %N, C:N ratios and isotope signatures of certain zooplankton and macrozoobenthos taxa. Their spatial variations were not tested due to the low number of replicates for individual zooplankton and macrozoobenthos taxa across space. However, spatial variability was accounted for in the analysis of seasonal differences of estuarine invertebrates by using spatial data (i.e. station number) as a random effect in the ANOVA analyses.

To determine if the elemental composition and stoichiometry of zooplankton and macrozoobenthos showed significant differences among taxa, one-way ANOVA using taxa as the factor was conducted. Seasonal and spatial variability on the nutrient content and ratio of estuarine invertebrates was accounted in the among-taxa variability analysis by using seasonal (i.e. seasons) and spatial (i.e. station number) data as random effects. All pair-wise comparisons were calculated using a Tukey HSD post-hoc test. The sequential Bonferroni test is sometimes used to adjust significance levels when multiple statistical tests are conducted. We did not apply a Bonferroni correction since there are several arguments against its use [27,28]. To reduce the risk of falsely rejecting true null hypotheses, we used a significance level of 0.01 for all tests since it reduces the rate of false positive by a factor of five. Homoscedasticity and normality were evaluated for all variables prior to the analyses using a Levene’s and Kolmogorov-Smirnov tests respectively, where necessary, variables were log_10_ transformed to fulfil the assumptions of parametric tests. Statistical analyses were conducted using the software IBM SPSS Statistics 21.

Diet proportions were calculated using the R package SIAR (Stable Isotope Analysis in R) [29]. We were interested in tracking the use of two food sources (susPOM and sedPOM) for zooplankton and macrozoobenthos over time in the two estuaries. The two sources are important components of the invertebrate’s diet (e.g. [30,31]) and an ubiquitous food source in estuaries. Zooplankton undergoes diurnal vertical migrations, and are therefore likely to ingest sedPOM in addition to susPOM. Zooplankton taxa in this analysis included *Acartia spp*. and *Pseudodiaptomus hessei* for both estuaries. For the Mysidaceae *Rhophalophthalmus terranatalis* (Mlalazi) and *Mesopodopsis africana (*Mpenjati), the suite of food sources was expanded from susPOM and sedPOM to include copepods to account for their partially carnivorous feeding mode [30]. Of the macrozoobenthos taxa, the preference for susPOM and sedPOM by *Apseudes digitalis*, *Dosinia hepatica*, *Macoma litoralis*, Spionidae and Cirratulidae was investigated in the Mlalazi Estuary, and of *Capitella capitata*, *Ceratonereis keiskama*, *Dendronereis arborifera* and Spionidae in the Mpenjati Estuary. The fractionation values used for the analyses were Δδ^15^N = 3.4‰ and Δδ^13^C = 1‰ [32]. As there was not enough biomass for spatial analysis, we concentrated on the seasonal variation of diet proportions. Our analysis of diet proportions thus did not account for finer scaled spatial resolution.

### Ethics statement

Sampling permits for scientific investigation were granted to the School of Life Sciences, University of KwaZulu-Natal, by the Department of Environmental Affairs and Tourism, Republic of South Africa. Permission for this study was obtained after registering the project titled “The ecosystem functioning of estuaries in KwaZulu-Natal (South Africa) and their influence on the nearshore coastal environment” with Ezemvelo KwaZulu-Natal Wildlife Provincial Authority.

## Results

### Environmental variables

At the Mlalazi Estuary, monthly rainfall varied from 11.0 mm in September 2010 to 230.4 mm in January 2011 (S1 Fig). Monthly rainfall varied from 18.2 mm in September 2010 to 223.5 mm in May 2011 during an abnormally wet winter on the Mpenjati Estuary (S1 Fig). A steady increase in monthly rainfall was observed from September 2010 to January 2011 in the catchment of both estuaries as expected during the rainy season. Sub-surface and bottom salinities were highest during September 2010 and lowest during February 2011 at the Mlalazi Estuary. A marked vertical gradient was observed in this estuary during February 2011 (S2 Fig). In the Mpenjati Estuary, lowest sub-surface and bottom salinity were recorded during November and September 2010 respectively, while highest sub-surface and bottom salinities were recorded during February 2011. A marked vertical salinity gradient was observed during November 2010 and May 2011 in the Mpenjati Estuary (S2 Fig).

### Variability of elemental content and stoichiometry of POM

The susPOM %C was higher compared to that of sedPOM in both estuaries (Table 1), with a seasonal mean of 2.5 ± 1.5 (SD) and 0.4 ± 0.3 (SD) at the Mlalazi and Mpenjati estuaries respectively, whereas sedPOM %C had a seasonal mean of 3.4 ± 2.8 (SD) and 0.3 ± 0.2 (SD) at the Mlalazi and Mpenjati estuaries respectively. Very low %N values were recorded for susPOM (mean <0.4) and sedPOM (mean <0.09) (Table 1).

**Table 1:** Elemental content and stoichiometry of taxa analyzed during this study and in other freshwater and marine global locations. Values represent mean ± SD, unless otherwise stated.

Taxa	Location	% C	% N	C:N ratio	References
susPOM	Baltic Sea	-	-	6.6–9.0	Walve and Larsson [49]
	Moji-Guaçu wetland, Brazil	-	-	6.6–11.0	Albuquerque and Mozeto [34]
	Mlalazi Estuary, South Africa	2.5 ± 1.5	0.4 ± 0.3	7.7 ± 1.7	**This study**
	Mpenjati Estuary, South Africa	3.4 ± 2.8	0.3 ± 0.2	9.6 ± 2.7	**This study**
sedPOM	C 53 and C 54, United States of America (U.S.A)	-	-	29–34	Cross et al. [40]
	Neuse, Pamlico and South Creek estuaries, U.S.A.	-	-	4.5–20.2	Matson and Brinson [56]
	Shuangtaizi wetland, China	-	-	16.2 ± 6.1	Zhang et al. [38]
	Great Ouse Estuary, England	-	-	5–22	Trimmer et al. [57]
	Pearl River Estuary, China	-	-	10.4 ± 1.3–15.2 ± 3.3	Yu et al. [58]
	Mlalazi Estuary, South Africa	1.0 ± 0.6	0.08 ± 0.04	13.8 ± 1.8	**This study**
	Mpenjati Estuary, South Africa	0.2 ± 0.2	0.01 ± 0.01	17.7 ± 3.7	**This study**
*Acartia* spp.	Baltic Sea	~ 46–51	~ 12–13	4.5–4.7	Walve and Larsson [49]
	Oslofjord, Norway	-	-	4.8 ± 0.7–8.7 ± 2.2	Gismervik [59]
	Sargasso Sea (copepods)	35.2–47.6	8.2–11.2	~ 5.04	Beers [47]
	Mlalazi Estuary	36.8 ± 8.7	8.7 ± 2.5	4.3 ± 0.6	**This study**
	Mpenjati Estuary (*A*. *natalensis*)	39.3 ± 5.6	9.9 ± 1.6	4.0 ± 0.1	**This study**
*Pseudodiaptomus* spp.	Mlalazi Estuary	49.7 ± 5.4	11.7 ± 1.1	4.3 ± 0.3	**This study**
	Mpenjati Estuary (*P*. *hessei*)	43.2 ± 5.3	10.4 ± 1.7	4.2 ±0.3	**This study**
Mysids	Sargasso Sea (mysids-euphasids)	35.4–43.4	9.4–10.5	~ 4.8	Beers [47]
	Mlalazi Estuary (*R*. *terranatalis*)	52.3 ± 1.5	14.3 ± 0.8	3.7 ± 0.1	**This study**
	Mpenjati Estuary (*M*. *africana*)	50.3 ± 3.0	13.1 ± 1.1	3.8 ± 0.2	**This study**
Other Crustaceans	35 streams, U.S.A. (5 taxa)	34.8 ± 1.7	7.4 ± 0.5	5.5 ± 0.3	Evans-White et al. [54]
	8 lakes, Canada (amphipods)	36.2 ± 2.2	7.3 ± 0.7	5.8 ± 0.6	Frost et al. [45]
	Lake Erken, Sweden (isopods)	-	-	5.4 ± 0.7	Liess and Hillebrand [41]
	Mlalazi Estuary (*A*.*digitalis*)	45.3 ± 3.2	10.8 ± 1.0	4.2 ± 0.2	**This study**
	Mlalazi Estuary (*P*. *blephariskios*)	41.9 ± 4.9	9.0 ± 1.3	4.7 ± 0.4	**This study**
Molluscs	35 streams, U.S.A. (11 taxa)	42.2 ± 3.6	9.6 ± 1.6	5.2 ± 0.7	Evans-White et al. [54]
	Lake Erken, Sweden (bivalves)	42.0 ± 1.3	10.3 ± 0.3	5.6 ± 0.9	Liess and Hillebrand [41]
	Antartic Sea (*Nacella concinna*)	48.8 ± 1.0 SE -49.4 ± 4.9 SE	10.6 ± 0.3 SE -12.6 ± 0.2 SE	4.5 ± 0.03 SE—5.4 ± 0.1 SE	Clarke [48]
	Mlalazi Estuary (*D*. *hepatica*)	45.7 ± 3.4	11.1 ± 1.3	4.1 ± 0.3	**This study**
	Mlalazi Estuary (*M*. *litoralis*)	44.1 ± 6.6	10.8 ± 1.9	4.1 ± 0.2	**This study**
Polychaetes	Antartic Sea (2 species)	51.1 ± 2.7 SE—53.1 ± 0.8 SE	10.9 ± 0.2 SE—11.3 ± 0.6 SE	5.3 ± 0.1 SE—5.9 ± 0.1 SE	Clarke [48]
	Mlalazi Estuary (Cirratulidae)	54.5 ± 8.1	9.4 ± 1.0	5.9 ± 1.3	**This study**
	Mlalazi Estuary (*Glycera* spp.)	53.6 ± 5.6	11.4 ± 1.7	4.9 ± 1.5	**This study**
	Mlalazi Estuary (*S*. *parva*)	50.3 ± 4.1	11.4 ± 1.2	4.8 ± 1.3	**This study**
	Mlalazi Estuary (Spionidae)	53.0 ± 11.2	9.5 ± 2.4	6.0 ± 2.2	**This study**
	Mpenjati Estuary (*C*. *capitata*)	49.1 ± 8.0	10.4 ± 1.9	4.8 ± 0.5	**This study**
	Mpenjati Estuary (*C*. *keiskama*)	49.7 ± 5.5	11.4 ± 1.1	4.4 ± 0.5	**This study**
	Mpenjati Estuary (*D*. *arborifera*)	48.0 ± 6.6	10.9 ± 1.7	4.5 ± 0.7	**This study**
	Mpenjati Estuary (Spionidae)	44.8 ± 5.8	9.9 ± 1.8	4.6 ± 0.5	**This study**

Elemental content of POM was very variable across seasons within and between estuaries (Table 2). Both C and N content of susPOM in the Mlalazi Estuary were lowest in a dry season month (September 2010) and highest in a wet season month (February 2011). In the Mpenjati Estuary, a pattern opposite to that of the Mlalazi was apparent with highest, instead of lowest, %C and %N for susPOM in the dry season month September 2010 and significantly lower values in wet season months. Significant spatio-temporal differences were recorded for the nutrient content of sedPOM in both estuaries, however, no clear seasonal trend (dry/wet season) was identified in both estuaries. Not only was POM elemental content significantly different in some seasons, but significant spatial differences were found for %C and %N for both the suspended and sediment particulate matter at the Mlalazi Estuary (Table 2). Higher concentrations of both C and N were measured in the upper reaches of the estuary (toward the river), compared to the lower reaches (toward the sea). The same trend was apparent from the Mpenjati Estuary for susPOM, but was reversed for N in sedPOM.

**Table 2:** Two-way ANOVA results for %C, %N and C:N ratio of suspended and sediment particulate matter in the study estuaries.

			susPOM		sedPOM	
Estuary	Component	Variables	*df*	*p*	*df*	*p*
Mlalazi	% C POM	Se	4, 58	<0.001	4, 58	<0.001
		St	3, 58	<0.001	3, 58	<0.001
		Se × St	12, 58	<0.001	11, 58	<0.001
Mlalazi	% N POM	Se	4, 58	<0.001	4, 58	<0.001
		St	3, 58	<0.001	3, 58	<0.001
		Se × St	12, 58	<0.001	11, 58	<0.001
Mlalazi	C:N POM	Se	4, 58	<0.001	4, 58	<0.001
		St	3, 58	0.018	3, 58	0.051
		Se × St	12, 58	0.031	11, 58	<0.001
Mlalazi	δ^13^C	Se	4, 53	<0.001	4, 58	<0.001
		St	3, 53	<0.001	3, 58	<0.001
		Se × St	11, 53	<0.001	11, 58	<0.001
Mlalazi	δ^15^N	Se	4, 53	<0.001	4, 58	<0.001
		St	3, 53	<0.001	3, 58	<0.001
		Se × St	11, 53	<0.001	11, 58	<0.001
Mpenjati	% C POM	Se	4, 43	<0.001	3, 26	<0.001
		St	2, 43	<0.001	2, 26	0.006
		Se × St	8, 43	0.218	4, 26	<0.001
Mpenjati	% N POM	Se	4, 43	<0.001	3, 26	0.001
		St	2, 43	<0.001	2, 26	0.001
		Se × St	8, 43	0.224	4, 26	<0.001
Mpenjati	C:N POM	Se	4, 43	<0.001	3, 26	0.291
		St	2, 43	0.758	2, 26	0.001
		Se × St	8, 43	<0.001	4, 26	0.095
Mpenjati	δ^13^C	Se	4, 41	<0.001	4, 23	0.075
		St	2, 41	0.001	2, 23	0.039
		Se × St	8, 41	<0.001	4, 23	0.135
Mpenjati	δ^15^N	Se	4, 41	<0.001	4, 23	0.151
		St	2, 41	0.421	2, 23	0.078
		Se × St	8, 41	0.003	4, 23	0.448

Abbreviations indicate seasons (Se), stations (St).

In both estuaries, susPOM had a lower C:N compared to sedPOM (Table 1). Significantly higher C:N of susPOM were measured in both the Mlalazi and Mpenjati during a dry season month (September 2010) compared to all other months. Similarly, sedPOM showed significantly higher C:N during the dry season months in the Mlalazi, but no significant seasonal difference in the Mpenjati Estuary (Table 2). Spatial differences of C:N in POM along the estuarine gradient were significant for susPOM, which was higher in the lower reaches of the Mlalazi compared to its upper reaches, but not in the Mpenjati (Table 2). C:N of sedPOM was significantly higher in the upper reaches of the Mpenjati compared to the middle and lower reaches, and no significant difference was apparent from the Mlalazi.

### Variability of elemental content and stoichiometry of consumers and homeostasis

Carbon content of invertebrates ranged from a mean of 36.8%C (± 8.8 SD) for *Acartia* spp. (Mlalazi) to a high of 54.5%C (± 8.1 SD) for Cirratulidae (Mlalazi) and 53.0%C (± 11.2 SD) for Spionidae (Mlalazi). Nitrogen content ranged from the lowest mean of 8.7% (± 2.6 SD) for *Acartia* spp. in the Mlalazi Estuary to a highest mean of 13.1% (± 1.1 SD) for *Mesopodopsis africana* in the Mpenjati Estuary (Table 1). Significant seasonal differences were absent from the elemental content of invertebrates, with one exception, which was the nitrogen content of the tanaid *Apseudes digitalis* at the Mlalazi Estuary. The latter was not related to the seasonal variations of the susPOM and sedPOM (Table 2).

In terms of C:N ratio, the C:N of the family Cirratulidae (C:N = 5.9 ± 1.3 SD) and Spionidae (C:N = 6.0 ± 2.2 SD) gave the highest mean overall, and *M*. *litoralis* the lowest (C:N = 4.1 ± 0.2 SD). Mean C:N ratios of invertebrates were lower than the POM C:N ratios (Table 1). Seasonal differences in C:N were also largely absent and only apparent for the copepod *Pseudodiaptomus* spp. (lowest and highest values during the dry season months May 2011 and September 2010 respectively) and the tanaid *A*. *digitalis* (highest value during the wet season month November 2010) in the Mlalazi Estuary.

Significant differences among taxa were only recorded for the nitrogen content of zooplankton taxa [F (5, 8.7) = 6.4, p = 0.009], where highest nitrogen content was exhibited by the mysids *Mesopodopsis africana* and *Rhopalophthalmus terranatalis*. No significant differences were recorded for the nutrient content or C:N ratio of macrozoobenthos taxa.

### Stable isotope signatures (δ^13^C, δ^15^N) and diet proportions

**Fig 2:**
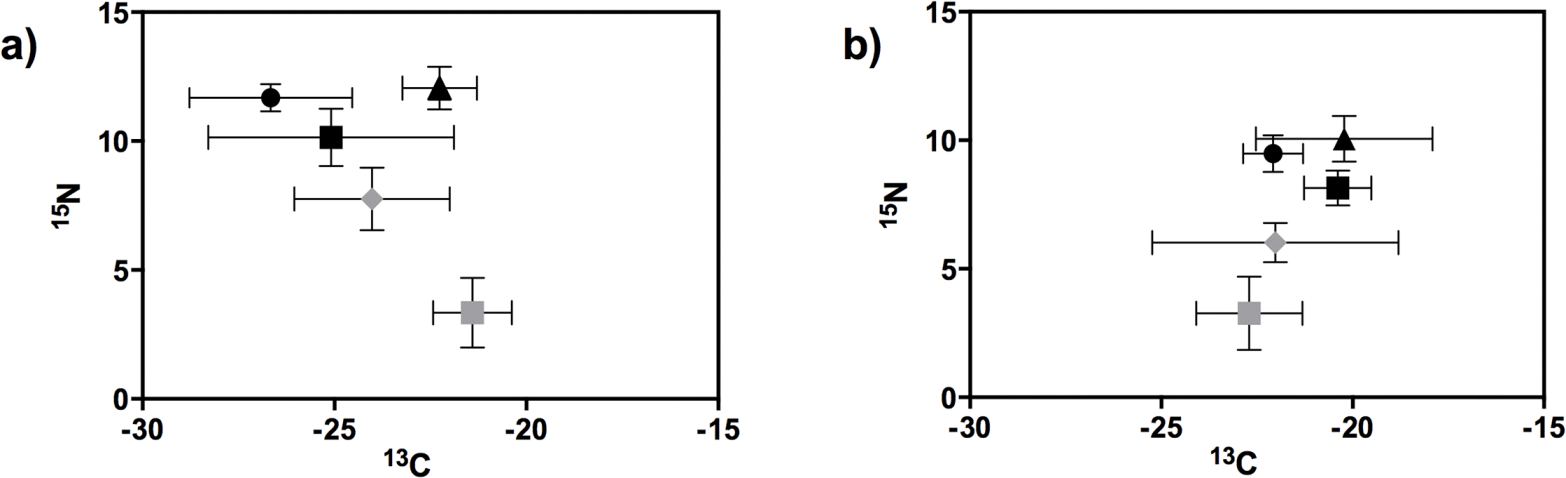
Mean (± SD) δ^13^C and δ^15^N of zooplankton species in the Mlalazi (right) and Mpenjati (left) estuaries. Color and shape indicate group or taxa (grey rhombus = susPOM, grey square = sedPOM, black circle = *Acartia* spp (a) and *Acartia natalensis* (b), black square = *Pseudodiaptomus* spp. (a) and *Pseudodipatomus hessei* (b), black triangle = *M*. *africana* (a) and *R*. *terranatalis* (b)).

**Fig 3:**
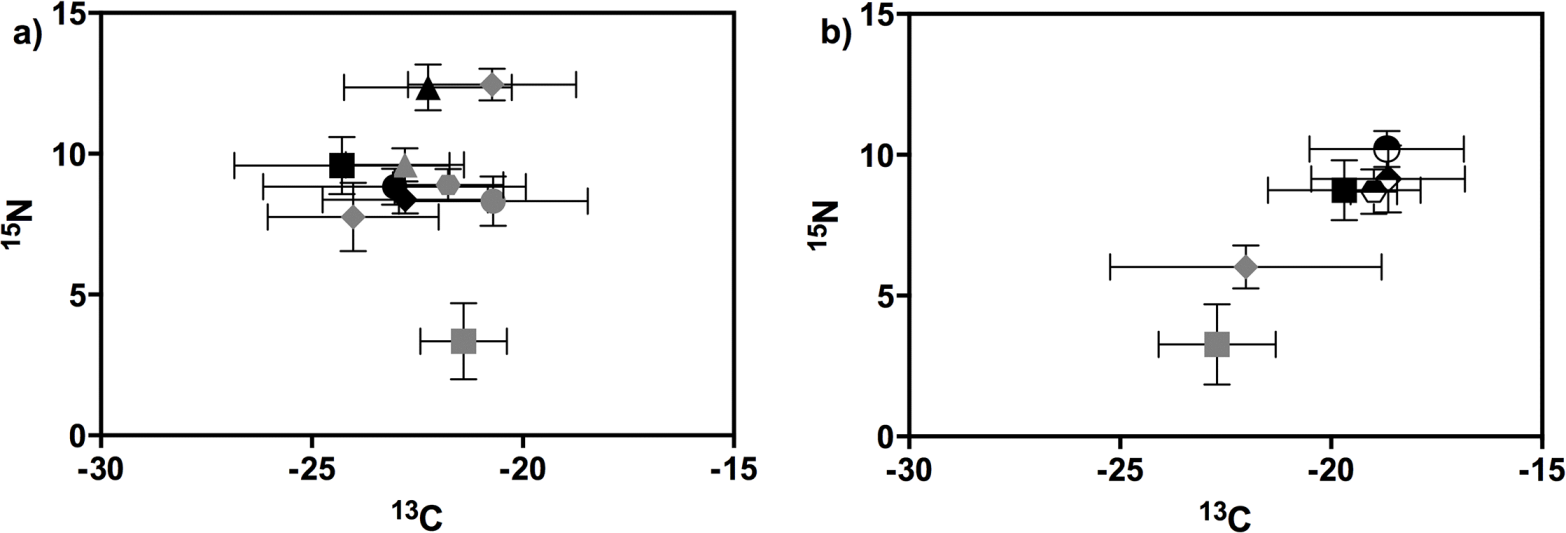
Mean (± SD) δ^13^C and δ^15^N of macrozoobenthos species in the Mlalazi (right) and Mpenjati (left) estuaries. Color and shape indicate group or taxa (grey rhombus = susPOM, grey square = sedPOM, black square = Spionidae, black rhombus = *P*. *blephariskios*, black triangle = *A*. *parva*, black circle = *A*. *digitalis*, grey rhombus = *Glycera* spp., grey triangle = Cirratulidae, grey circle = *M*. *litoralis*, grey hexagon = *D*. *hepatica*, half rhombus = *D*. *arborifera*, half circle = *C*. *keiskama*, half hexagon = *C*. *capitata*).

Little variation was recorded for the mean susPOM δ^13^C and δ^15^N values between the Mlalazi (δ^13^C = -24.02 ± 2.02 ‰ SD and δ^15^N = 7.75 ± 1.22 ‰ SD) and Mpenjati estuaries (δ^13^C = -21.51 ± 3.41 ‰ SD and δ^15^N = 6.02 ± 0.77 ‰ SD) (Figs 2 and 3). Mean sedPOM δ^13^C (Mlalazi = -21.40 ± 1.02 ‰ SD; Mpenjati = -22.73 ± 1.38 ‰ SD) and δ^15^N (Mlalazi = 3.34 ± 1.35 ‰ SD; Mpenjati = 3.12 ± 1.34 ‰ SD) were also similar in both estuaries. Whereas susPOM had similar δ^13^C signatures to sedPOM, the δ^15^N of susPOM was considerably higher than that of sedPOM in both estuaries (Figs 2 and 3). Significant spatio-temporal differences were found for δ^13^C and δ^15^N in both susPOM and sedPOM in the Mlalazi Estuary (Table 2), however no clear seasonal trend was observed with high and low values recorded during both dry and wet seasons. In the Mpenjati Estuary, significant spatio-temporal differences were recorded for δ^13^C and δ^15^N of susPOM, but not for sedPOM (Table 2). More enriched δ^13^C and δ^15^N of SusPOM were recorded during the dry season month September 2010, with the opposite pattern during the wet season month February 2011.

The δ^13^C of zooplankton species in the Mlalazi Estuary was usually more depleted, and the δ^15^N signatures more enriched, than of those in the Mpenjati Estuary (Fig 2). The most depleted δ^13^C was that of *Acartia* spp. (δ^13^C = -26.56 ± 2.12 ‰ SD) and the most enriched δ^13^C was *M*. *africana* (δ^13^C = -20.22 ± 2.3 ‰ SD). The lowest δ^15^N signatures was that of *P*. *hessei* (δ^15^N = 8.12 ± 0.69 ‰ SD) and the highest was *R*. *terranatalis* (δ^15^N = 12.06 ± 0.83 ‰ SD). The δ^13^C signatures among macrobenthic species ranged from the polychaetes *D*. *arborifera* (-18.65 ± 1.82 ‰ SD) and *C*. *keiskama* (-18.68 ± 1.83 ‰ SD) to Spionidae (-24.29 ± 2.55 ‰ SD) (Fig 3). The lowest δ^15^N signatures were recorded for the bivalve *M*. *litoralis* (8.32 ± 0.88 ‰ SD) and the crab *P*. *blephariskios* (8.37 ± 0.49 ‰ SD), and the highest δ^15^N signatures for the polychaetes *S*. *parva* (12.35 ± 0.82 ‰ SD) and *Glycera* spp. (12.46 ± 0.57 ‰ SD) (Fig 3). The δ^15^N signatures of most macrobenthic species, without taking in consideration the carnivores, fell within a range of 1.9 ‰. Seasonal differences in δ^13^C were detected for *Pseudodiaptomus* spp. in the Mlalazi, with more depleted carbon signatures in May 2011. Of the macrozoobenthos taxa, only the polychaete *Dendronereis arborifera* exhibited significant temporal variations on their δ^15^N. *D*. *arborifera* had more enriched signatures during May 2011.

**Fig 4:**
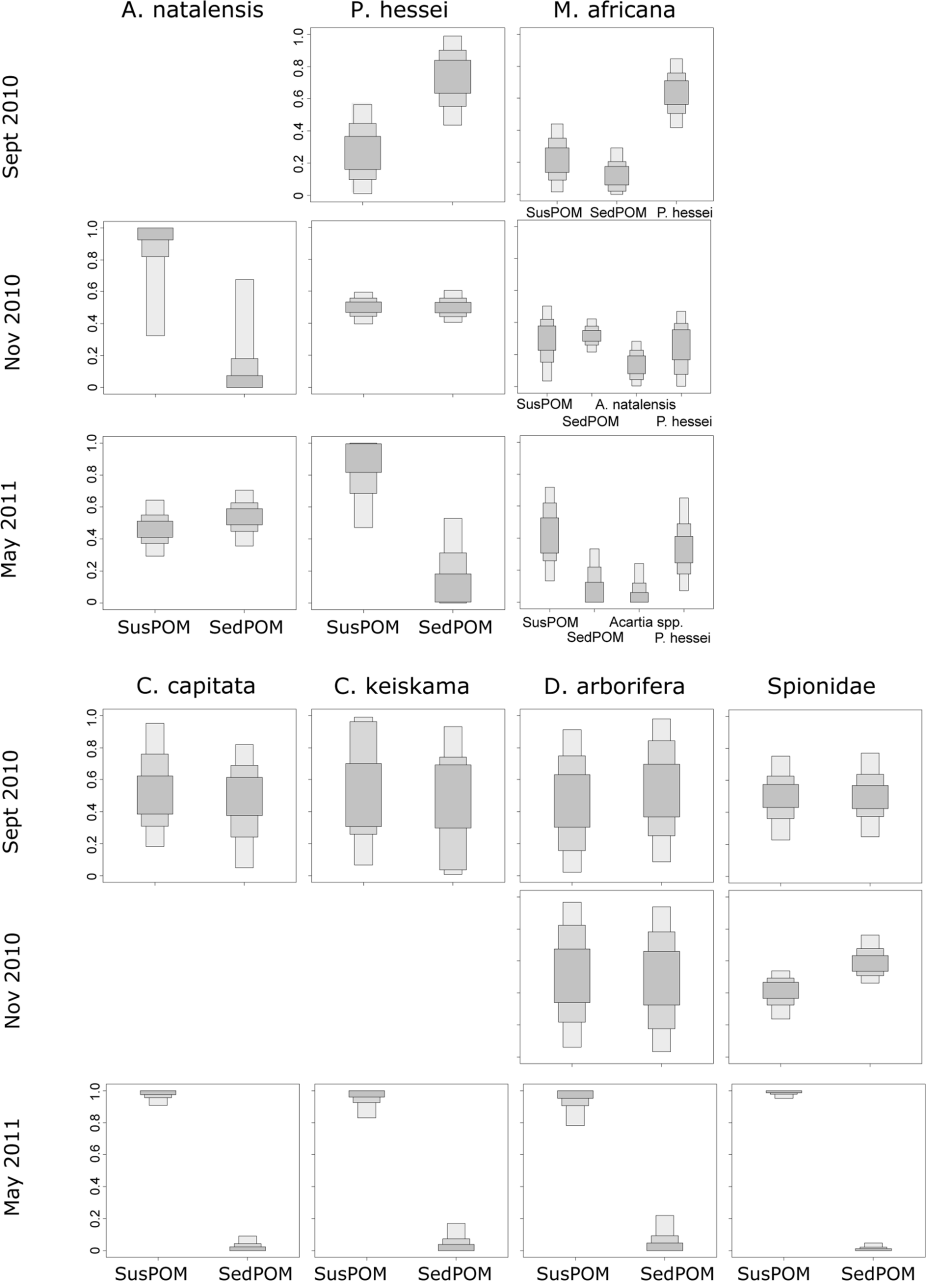
Diet proportions of zooplankton and macrozoobenthos invertebrates in the Mpenjati Estuary. Food sources are suspended (susPOM) and sediment (sedPOM) particulate organic matter. *Mesopodopsis* has additional food sources: *Acartia natalensis* and *Pseudodiaptomus hessei*.

**Fig 5:**
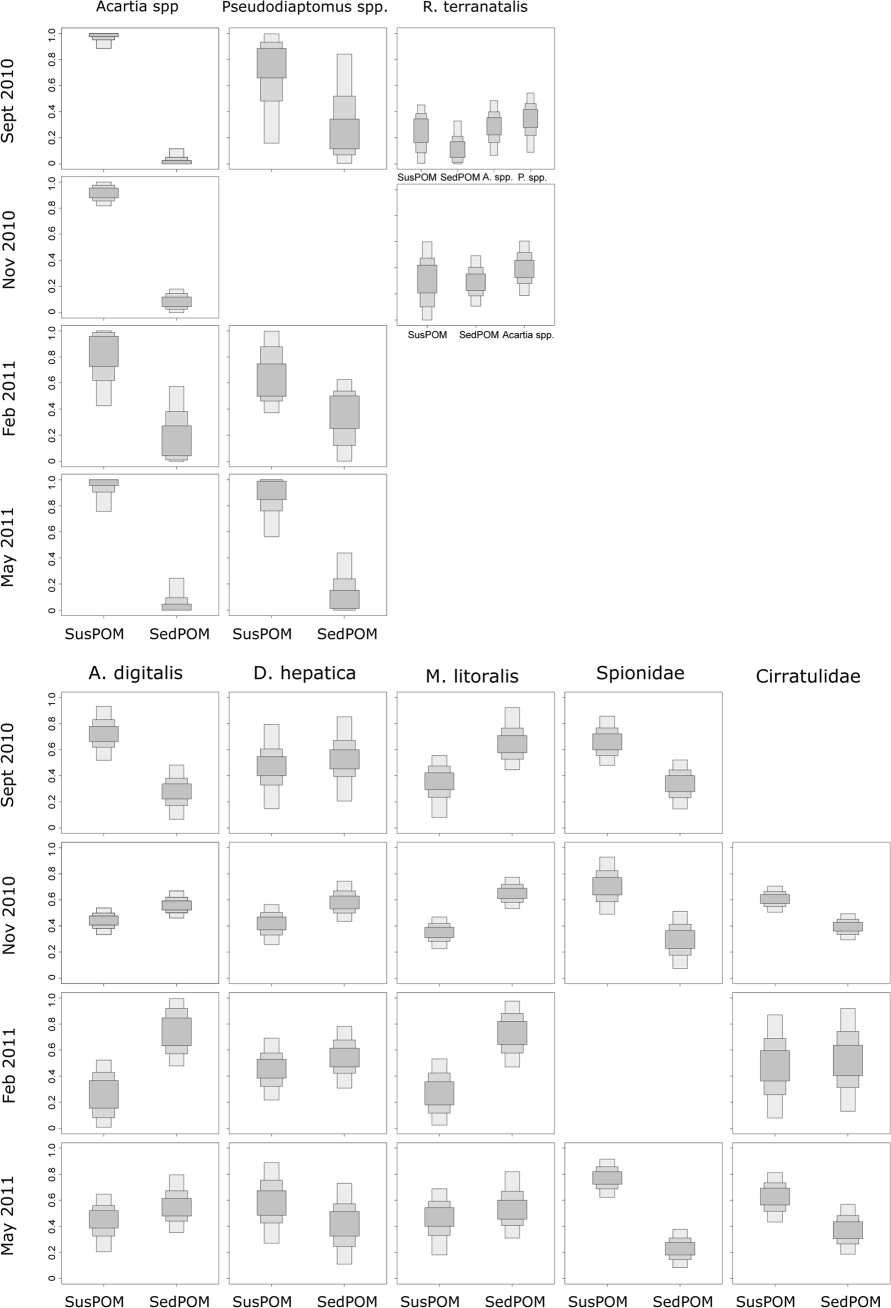
Diet proportions of zooplankton and macrozoobenthos invertebrates in the Mlalazi Estuary. Food sources are suspended (susPOM) and sediment (sedPOM) particulate organic matter. *Rhopalophthalmus* has additional food sources: *Acartia spp*. and *Pseudodiaptomus* spp.

Diet composition of invertebrates was analyzed to ascertain the different utilization of suspended and sediment detritus, and of the mysid species by adding copepods as a diet item. The diet proportions were investigated for all four seasons for the Mlalazi Estuary, but only for three seasons in the Mpenjati Estuary. In Feburary 2011, the difference between the isotope values of the sources (susPOM, sedPOM) were too small (<2‰) to warrant analysis [33]. There were seasonal differences apparent for the Mpenjati Estuary, where during the abnormally wet dry season month (May 2011), after a breach, the susPOM was more important as a food source compared to sedPOM for all zooplankton and benthic invertebrates, except for *Acartia spp*. (Fig 4). In comparison, in the dry season month September 2010 and wet season month November 2010, susPOM and sedPOM were about equally important as a food source for the benthic species, with no distinct pattern for the zooplankton species except that sedPOM was never the preferred source over SusPOM. In the Mlalazi Estuary no seasonal pattern for the utilization of POM was apparent for the mysid *R*. *terranatalis* and the benthic taxa, and the importance of the two food sources in relation to each other depended on taxon (Fig 5). The two copepod taxa, *Acartia spp*. and *Pseudodiaptomus spp*., mostly preferred susPOM over sedPOM in the Mlalazi throughout all seasons.

Moreover, the same taxa did not always prefer the same food source in both estuaries. *Acartia* spp. in the Mlalazi Estuary preferred susPOM over sedPOM (Fig 5), whereas this was not apparent from *A*. *natalensis* in the Mpenjati Estuary (Fig 4). *Pseudodiaptomus spp*. overall preferred susPOM over sedPOM in the Mlalazi Estuary, but in the Mpenjati Estuary *P*. *hessei* showed such preference only in February and May 2011. The mysids *R*. *terranatalis* and *M*. *africana* preferred susPOM over sedPOM in most seasons and only on one occasion was a clear preference apparent for copepods over POM (September 2010, Figs 4 and 5). Of the macrozoobenthos species, only Spionidae showed a clear preference of susPOM over sedPOM in the Mlalazi Estuary. The other taxa showed variable, or no, preference (Figs 4 and 5).

## Discussion

The analysis of elemental content (C, N), stoichiometry (C:N) and diet composition in relation to seasonal variations of river inflow and rainfall in the temporarily open/closed Mpenjati Estuary and permanently open Mlalazi Estuary revealed a large influence of river inflow on suspended organic matter and to a lesser extent on sediment organic matter. River inflow had a minimal influence on the nutrient dynamics of invertebrates, its influence was mainly evident as a change in consumers’ diet after an extreme event. Although different behavior of elemental content across seasons or contrasting behavior between the two estuaries was apparent, a few patterns did emerge. For instance, whereas C:N of suspended particulate matter was significantly lower during wet season months in both estuaries as expected, elemental content was lowest during dry season months in the Mlalazi Estuary, but highest during dry seasons months in the Mpenjati Estuary. These results are in agreement with previous findings, which reported significantly lower C:N and C:P of suspended particulate matter during the wet season as a result of catchment wash-out from agriculturally derived N and P material or the increased nutrient input to estuaries arising from rainfall based catchment run-off [34,35]. The higher elemental content of the temporarily open/closed Mpenjati Estuary during the dry season month may have been a result of the less scouring and disturbance during this phase, which is also characterized by high biomass of primary producers and consumers (e.g. [19,36]).

Compared to susPOM, the stoichiometry of the sedPOM revealed more variability within and between the two estuaries, and its elemental content did not show a distinct seasonal pattern. Lower nutrient content of sedPOM was expected during the wet season in both systems due to the increased river inflow, scouring and flushing of the more organically rich surface sediments to the sea. However, low or high nutrient content of sediment particulate matter were not associated to a particular season (dry/wet) for both estuaries. This suggests that the elemental composition of sediment particulate matter was not influenced by seasonal changes in river inflow during our study. The C:N of sedPOM at the Mlalazi Estuary was significantly higher during the dry season months as expected due to the lower exchange of carbon and other nutrients between the estuary and adjacent environments (e.g. decreased riverine inputs) during this season [37,38]; whereas no seasonal variations in the C:N ratio of sedPOM were recorded at the Mpenjati Estuary despite the reduced freshwater and nutrient input to the system during the dry season (closed phase). This suggest that the nutrient quality of sedPOM is less dependent on river flow and open/closed mouth conditions in temporarily open/closed estuaries, probably because of the frequent disturbances (i.e. flushing and scouring of the sediment) experienced in these systems. Due to the relatively widely spaced seasonal sampling regime, the effect of such extreme events could not be thoroughly investigated within our scope. The C:N ratio of the sedPOM from theS study estuaries fell within the range presented in other freshwater and marine systems (e.g.[39–41])

Previous studies have reported a marked variability in the elemental composition and ratios of benthic and zooplanktonic invertebrates [42–46], this variability was evidenced as changing with locations, seasons and as a response to changes in the stoichiometry of their food sources. Higher variability of elemental content and C:N throughout the study period was apparent for POM in comparison to invertebrates. In our study, no consistent seasonal pattern in the nutrient content and ratio of estuarine invertebrates was found and the seasonal variations in their stoichiometry did not follow the stoichiometric changes in their food sources (i.e. susPOM or sedPOM composition, and also copepods for mysids). These findings are in agreement with Andersen and Hessen [47] who reported no correspondence between the seasonal variations in nutrient content among zooplankton species in a Norwegian lake. In terms of benthic consumers, variable results regarding a stoichiometry relationship between food sources and consumers are reported in the literature, i.e. Cross et al. [43] and Liess and Hillebrand [44] found a relationship between macrozoobenthos stoichiometry and potential food sources (periphyton in their case), but other studies have reported a lack thereof [48,49] similar to our study areas. In our case it is also possible that analyzing POM masked the importance of more nutritious food sources contained therein, such as benthic algae, bacteria and microfauna. Similarly, the possible contribution of different food sources in the analysis of POM might explain the seasonal differences recorded. Also, the seasonal sampling regime did not allow for close examination of a lagged response of consumer stoichiometry to that of their food sources. The nutrient content of the zooplankton and macrozoobenthos in this study fell within the range presented for other invertebrates in freshwater and marine systems (e.g. [50–53]) (Table 1).

Only two taxa (the copepod *Pseudodiaptomus* spp. and the tanaid *A*. *digitalis*) exhibited significant differences in their C:N ratio among seasons suggesting that most consumers maintain a tight homeostasis despite the variations in their food source (POM) and physical environment. The maintenance of homeostasis can partially be attributed to the ability of the consumers to excrete excess carbon, and by supplementing their diet with nutrient richer sources such as microalgae (not investigated in this study [30]. The invertebrates investigated not only maintained tight homeostasis and as such provided a relatively stoichiometrically stable food source, but also concentrated the elemental content between POM and its consumers by 10–100 fold. This wide range was mainly driven by the more variable stoichiometry of the POM source. Although the general understanding from ecological pyramids is that the transfer efficiency between food source and consumer is in the range of 10%, when investigating individual ecosystems and especially different elements, it can quickly be discerned that transfer efficiencies are variable (e.g. [54,55,56]). Thus, depending on consumer needs and resource supply, consumers adjust their proportional intake of the required elements.

Variability in the elemental composition and stoichiometry among aquatic invertebrates have been mainly related to feeding guilds and phylogeny (e.g. [57,58,59]). In terms of zooplanktonic taxa, the herbivorous/detritivorous copepods *Acartia* spp. showed lowest nutrient content in both estuaries and the predatory mysids *M*. *africana* and *R*. *terranatalis* the highest among zooplankton taxa. These mysids also had the lowest C:N ratio among zooplankton taxa. The polychaetes *Glycera* spp., and Cirratulidae had the highest carbon content among macrozoobenthos taxa. Only one other study was found in the literature that analyzed the elemental composition of polychaetes [51], who reported comparable findings of high carbon and nitrogen content for two species of polychaetes in the Antarctic Sea (Table 1). The polychaetes *S*. *parva*, *C*. *keiskama* and *Glycera* spp. had the highest nitrogen content among the studied zoobenthic taxa. The families Glyceridae (*Glycera* spp.) and Pilargidae (*S*. *parva*) are primarily carnivores while the Nereidae (*C*. *keiskama*) can act as carnivore, filter feeder or surface deposit feeder [31]. Their comparatively highest δ^15^N signatures confirmed that *S*. *parva* and *Glycera* spp. are predators. Previous studies have reported higher N content in predators than detritivores and herbivores [43,51,57], and our results from estuarine systems provided support for this theory. The deposit feeding crab *P*. *blephariskios* showed lowest carbon and nitrogen content among benthic taxa. Fagan et al. [57] suggested that herbivores have a lower nitrogen content than carnivores because they evolved to a lower dependence on N due to their food sources being N limited. The low N content of detritivores [59] could be explained by the low nutrient quality of their food sources [57] highlighting that the nutrient content of a consumer may be influenced by food source quality [57,59]. Furthermore, Evans-White et al. [58] reported a lower nutrient content for crustaceans when compared to molluscs. Our results agreed with their findings in that crustaceans with detritivore/herbivore feeding mode had the lowest nutrient content among taxa. Likewise, the deposit feeders Cirratulidae and Spionidae had the highest C:N ratio among taxa. Most analyzed taxa fell within the suspension or deposit feeders category (e.g. [31]), thus, it is possible that the differences in elemental ratio found among invertebrates were related to factors other than feeding guild such as phylogeny.

In summary, seasonal variations in river inflow influenced the C and N dynamics of suspended POM, less so for sediment POM, and riverine influence was largely absent for zooplankton and macrozoobenthic taxa in the two estuaries. Our results suggests that feeding mode was more important in explaining the nutrient dynamics of estuarine invertebrates compared to river flow. River flow did however influence the proportional reliance of invertebrates on suspended or sediment POM, which was most clearly apparent in the taxa from the temporarily open/closed estuary (Mpenjati) just after it had breached in an abnormally wet winter (May 2011). The first order consumers accrued the comparatively low nutrient content of their POM food sources by 10 to 100 fold. As it is an energy consuming process to ingest sufficient amounts of the limiting nutrient and expel excess carbon to arrive at the preferred C:N, one would suspect that the more nutrient enriched food source (in this case susPOM) was preferably consumed, however this was not always the case. Whichever the preferred food source, first order consumers managed to keep a relatively tight homeostasis and, as has previously been recognized, play a key functional role in ecosystems by providing preferable nutrient conditions for carnivorous feeders and omnivores at higher trophic levels in food webs.

Viewing our findings in the context of climate change scenarios predicting a higher number of days with rain and higher rainfall for the region, it is anticipated that the main type of estuaries along the coast (i.e. TOCE), will open and flush out more frequently to the sea compared to the past. Following the pattern seen from the TOCE Mpenjati, the increased importance of nutrient richer suspended POM as a food source during higher rainfall and river flow scenarios, may influence the energy expenditure of first order consumers to achieve their preferred stoichiometry. However, as the entire dry-wet cycle may change, more and more severe rainfall may include unprecedented consequences for elemental content and stoichiometry of invertebrates.

## Supporting Information

S1 Fig Total monthly rainfall in the catchment of the Mlalazi and Mpenjati estuaries. Colors indicate estuary (black = Mlalazi and grey = Mpenjati). Data corresponds to station 478, Empangeni and station 101, Southbroom respectively- South African Sugarcane Research Institute (SASRI).
(TIFF)

S2 Fig Mean (± SD) salinity for the Mlalazi (a) and Mpenjati (b) estuaries. Colors indicate seasons (black = September 2010, grey = November 2010, dark grey = February 2011 and light grey = May 2011).
(TIFF)
